# Dose–volume parameters and local tumor control in cervical cancer treated with central-shielding external-beam radiotherapy and CT-based image-guided brachytherapy

**DOI:** 10.1093/jrr/rrz023

**Published:** 2019-05-21

**Authors:** Shohei Okazaki, Kazutoshi Murata, Shin-ei Noda, Yu Kumazaki, Ryuta Hirai, Mitsunobu Igari, Takanori Abe, Shuichiro Komatsu, Takashi Nakano, Shingo Kato

**Affiliations:** 1 Department of Radiation Oncology, Gunma University Graduate School of Medicine, Showa-machi, Maebashi, Gunma, Japan; 2 Department of Radiation Oncology, Saitama Medical University International Medical Center, 1397-1 Yamane, Hidaka, Saitama, Japan

**Keywords:** uterine cervical cancer, radiotherapy, 3D image-guided brachytherapy, dose–volume parameter

## Abstract

Definitive radiotherapy for cervical cancer consists of external-beam radiotherapy (EBRT) and brachytherapy. In EBRT, a central shield (CS) reduces the dose to the rectum and bladder. The combination of whole-pelvic (WP)- and CS-EBRT and brachytherapy is the standard radiotherapy protocol in Japan. Despite clinical studies, including multi-institutional clinical trials, showing that the Japanese treatment protocol yields favorable treatment outcomes with low rates of late radiation toxicities, dose–volume parameters for the Japanese treatment protocol remain to be established. We conducted a retrospective dose–volume analysis of 103 patients with uterine cervical cancer treated with the Japanese protocol using computed tomography–based adaptive brachytherapy. The 2-year overall survival and 2-year local control rates according to FIGO stage were 100% and 100% for Stage I, 92% and 94% for Stage II, and 85% and 87% for Stage III–IV, respectively. Late adverse effects in the rectum and bladder were acceptable. Receiver operating characteristic analysis discriminated recurrence within the high-risk clinical target volume (HR-CTV) (*n* = 5) from no local recurrence (*n* = 96), with the optimal response obtained at a dose of 36.0 Gy_EQD2_ for HR-CTV D90 and 28.0 Gy_EQD2_ for HR-CTV D98. These values were used as cut-offs in Fisher exact tests to show that high HR-CTV D90 and HR-CTV D98 doses for brachytherapy sessions were significantly associated with tumor control within the HR-CTV. These data suggest a contribution of brachytherapy to local tumor control in WP- and CS-EBRT and brachytherapy combination treatment, warranting validation in multi-institutional prospective studies.

## INTRODUCTION

The standard radiotherapy (RT) protocol for uterine cervical cancer is the combination of external-beam radiotherapy (EBRT) to the pelvis and brachytherapy (BT) [1]. BT is a critical component in the treatment of cervical cancer [2]. Three-dimensional image-guided BT (3D-IGBT) using computed tomography (CT) or magnetic resonance imaging (MRI) enables delivery of sufficiently high doses to the cervical tumor while minimizing the doses to organs at risk (OARs), such as the rectum, sigmoid colon, and bladder, contributing to favorable local tumor control with reduced adverse events [3–9]. The use of 3D-IGBT in the treatment of cervical cancer is increasing worldwide [10–13].

The Group Européen de Curiethérapie–European Society for Therapeutic Radiology and Oncology (GEC-ESTRO) working group for gynecologic BT provides recommendations on 3D-IGBT for cervical cancer. The recommendations cover the concepts, terms and implications of 3D-IGBT, including definitions of the high-risk (HR) and intermediate-risk (IR) clinical target volumes (CTVs) of BT, 3D image-based treatment planning, dose–volume parameters of the CTVs and OARs, and recording and reporting of dose–volume parameters, among others [14, 15]. Several investigators have demonstrated a positive relationship between the dose–volume parameters of HR-CTV, such as dose covering 90% and 98% of the HR-CTV (D90 and D98) according to the GEC-ESTRO recommendations, and the probability of tumor control [16–18]. Studies suggest that the dose–volume parameters of the HR-CTV are important factors in local tumor control.

In EBRT for cervical cancer, a central shield (CS) is used to reduce the doses to the rectum and bladder, because these organs may be irradiated with very high doses of BT, potentially causing severe late radiation toxicities. The combination of whole-pelvic (WP)- and CS-EBRT and BT was established in Japan more than four decades ago as the standard RT protocol [19, 20]. In the USA or Europe, CS-EBRT is rarely used, whereas 45–50 Gy of WP-EBRT and 4–5 sessions of BT are commonly used for locally advanced cervical cancer. By contrast, the combination of 30–40 Gy of WP-EBRT, 20–10 Gy of CS-EBRT and 3–4 sessions of BT is commonly used in Japan. Many clinical studies, including several multi-institutional clinical trials, have demonstrated that the Japanese treatment protocol yields favorable treatment outcomes with low rates of late radiation toxicities [19–24]. The GEC-ESTRO working group have recommended that the accumulated dose–volume parameters for HR-CTV D90 and D98 should be estimated by simply adding the biologically equivalent dose of the WP-EBRT to that of every BT session, whereas the dose of the CS-EBRT should be excluded from the estimate [15]. Therefore, the estimated dose–volume parameters in the Japanese protocol are considered to be significantly lower than the actual irradiated dose [25]. Hence, how to accurately estimate the dose–volume parameters of HR-CTV when using CS-EBRT remains controversial [26, 27].

Here, we conducted a retrospective study of patients with cervical cancer treated with definitive RT or concurrent chemoradiotherapy (CCRT) using CT-based 3D-IGBT to determine the effective dose–volume parameters of the HR-CTV and the optimum dose for local tumor control in patients treated with WP- and CS-EBRT and BT.

## MATERIALS AND METHODS

### Patient selection

Cervical cancer patients who were treated with RT or CCRT at Saitama Medical University International Medical Center between June 2013 and October 2015 were enrolled in the study according to the following criteria: (i) histologically confirmed previously untreated carcinoma of the uterine cervix; (ii) 20–90 years of age; (iii) thoroughly evaluated by CT, MRI and a gynecological examination at pre-treatment time points; (iv) International Federation of Gynecology and Obstetrics (FIGO) Stage IB1–IVA; (v) treated with definitive RT or CCRT using CT-based 3D-IGBT; and (vi) available dose–volume histogram (DVH) data.

Patients who had para-aortic lymph node metastasis at the start of treatment were excluded from the study.

The present study complied with the standards of the Declaration of Helsinki. The study was approved by the institutional review board of Saitama Medical University International Medical Center (Registration number: 17–163).

### Radiotherapy

Radiotherapy consisted of EBRT and BT. EBRT was delivered to the pelvis with 10 MV X-rays. The CTV for the EBRT included the primary tumor, whole uterus, bilateral parametria, at least the upper half of the vagina, and pelvic lymph nodes (common-, internal-, and external iliac, obturator, and presacral lymph nodes). Three-dimensional conformal RT with anterior–posterior and two lateral portals was used for the treatment. EBRT was administered at a dose of 1.8–2.0 Gy/fraction and 5 fractions/week. After the delivery of 20 Gy (for Stage IB1 or IIA1) or 30–40 Gy (for more advanced stages of disease) of the WP, a 3 cm-wide CS was inserted into the pelvic field, and an additional 30–10 Gy was delivered to the pelvic sidewalls using anterior–posterior parallel-opposed portals, resulting in a total dose of 50 Gy. When a patient had bulky lymph node(s), an additional 6–10 Gy was delivered to the lesion(s) to boost the dose to 56–60 Gy.

BT was initiated after WP-EBRT and was performed weekly in 3–4 sessions in total. A high-dose rate ^192^Ir source was used for the treatment. Intracavitary (IC) BT or the combination of IC and interstitial (IS) BT was performed to adapt to the tumor volume. Use of the IS technique was considered for the patients with a large tumor and/or asymmetric lateral extension. CT-based 3D-IGBT was performed in every BT session. The HR-CTV and OARs (including the rectum, sigmoid colon, bladder, and small bowel) were delineated on CT images, with reference to MRI images acquired at diagnosis and just before the first session of BT and the findings of the gynecological examination by both gynecological and radiation oncologists. All radiation doses were biologically converted to equivalent doses in 2 Gy (EQD2) by the linear quadratic model using an alpha/beta ratio of 10 Gy for HR-CTV and 3 Gy for OARs. Regarding the dose–volume parameters, the D90 and D98 of the HR-CTV and the D2cm^3^ of the rectum, sigmoid colon, and bladder were calculated, recorded, and reported at every BT session. BT was planned for delivering the doses according to the following criteria: (i) planning aim dose to the HR-CTV D90 ≥6 Gy at each BT session, and the dose constraints to the OARs of the whole RT; (ii) D2cm^3^ of the rectum and sigmoid colon ≤70 Gy_EQD2_ and D2cm^3^ of the bladder ≤ 90 Gy_EQD2_; (iii) when the dose constraints of the HR-CTV and OARs were not achieved simultaneously, the constraints of the OARs were prioritized.

Radiotherapy was withheld if patients developed Grade 4 hematological toxicities or Grade 3–4 non-hematological toxicities as assessed by Common Terminology Criteria for Adverse Events (CTCAE) ver. 4.0 [28]. Radiotherapy was resumed when the toxicities recovered to Grade 2.

### Chemotherapy

Patients with T1b1N0 or T2a1N0 disease were treated with RT alone. Patients received weekly administration of cisplatin concurrently with EBRT if treatment was considered feasible based on their general conditions and organ functions. Patients who were considered unable to tolerate cisplatin because of old age (basically 75 years or older), and/or insufficient organ function received monthly administration of nedaplatin. Chemotherapy was withheld in patients showing a white blood cell count of <3000/mm^3^, platelet count <75 000/mm^3^, fever >38°C, or Grade 3–4 non-hematological toxicities. Chemotherapy was resumed when the toxicities recovered to Grade 1.

### Follow-up

Patients were followed up every 1–3 months for the first 2 years and every 3–6 months from the third year after completion of the treatment. Tumor status and adverse events were assessed using patient interviews, physical and gynecological exams, and blood tests. Patients underwent CT and/or MRI at 1 month after treatment to evaluate the therapeutic effects, and every 6–12 months thereafter. Recurrent disease was confirmed by biopsy whenever possible.

Treatment failure was classified into pelvic and extrapelvic failures. Pelvic failure was subclassified into local recurrence and regional lymph node recurrence. Local control (LC) duration was defined as the period from the date of initiation of RT to the date of diagnosis of local recurrence or the date of the most recent follow-up. Overall survival (OS) duration was defined as the period from the date of RT initiation to the date of death from any cause or the date of the most recent follow-up.

Late adverse events were defined as adverse events emerging after the 91st day from completion of RT and were graded according to the CTCAE version 4.0.

### Statistics

Statistical analyses were performed using SPSS v. 25.0 (IBM, Illinois, USA). OS and LC rates were calculated by the Kaplan–Meier method and compared by the log-rank test. Significant prognostic factors for local tumor control were identified by univariate analysis of the correlation of LC with various clinicopathological factors (age, T factor, initial tumor size, histology, and the use of chemotherapy) and dose–volume parameters of RT (dose of WP-EBRT and the cumulative HR-CTV D90 and D98 of all BT sessions and those of WP-EBRT + BT) using Fisher’s exact test. The cut-off values for dose–volume parameters related to LC were determined by receiver operating characteristic (ROC) analysis. *P* < 0.05 was considered statistically significant.

## RESULTS

### Patient characteristics and treatment

The present study enrolled 103 patients with uterine cervical cancer who met the eligibility criteria. The characteristics of the patients and treatment are summarized in Table 1. Regarding EBRT, a total dose of 39.6–55.0 Gy (median, 50.0 Gy) was delivered. A CS was inserted into the pelvic field after delivery of 19.8–45.0 Gy (median, 30.0 Gy). Regarding BT, a total of 3–5 (median, 4) sessions were performed. Nine patients received combination treatment with IC- and IS-BT. The median HR-CTV D90 and D98 of all BT sessions were estimated at 40.5 Gy_EQD2_ and 31.4 Gy_EQD2_, respectively. The median HR-CTV D90 and D98 of the whole RT, which were calculated by adding the doses of WP-EBRT and those of all BT sessions, were estimated at 74.2 Gy_EQD2_ and 65.1 Gy_EQD2_, respectively.

**Table 1.** Patient and treatment characteristics

Characteristics (103 patients)	
Age (*n*)	Median 64 years old (range: 29–85)
FIGO stage (*n*)	
IB1	7
IB2	4
IIB	50
IIIA	3
IIIB	33
IVA	6
Lymph node metastasis (*n*)	
Positive	39
Negative	64
Histologic subtype (*n*)	
SCC	89
AD	10
AdSq	3
SM	1
Tumor size (*n*)	
<4 cm	23
4–6 cm	50
>6 cm	30
EBRT	
Total dose	Median 50 Gy (range: 39.6–55)
WP-EBRT	Median 30 Gy (range: 19.8–45)
CS-EBRT	Median 20 Gy (range: 5.4–30)
BT	
IC-BT (*n*)	94
IC+IS-BT (*n*)	9
HR-CTV D90	Median 40.5 Gy_EQD2_ (range: 25.9–55.9)
HR-CTV D98	Median 31.4 Gy_EQD2_ (range: 18.3–45.4)
WP-EBRT + BT	
HR-CTV D90	Median 74.2 Gy_EQD2_ (range: 56.4–90.9)
HR-CTV D98	Median 65.1 Gy_EQD2_ (range: 46.8–81.0)
Chemotherapy (*n*)	76
CDDP	66
NDP	10

SCC = squamous cell carcinoma, AD = adenocarcinoma, AdSq = adenosquamous cell carcinoma, SM = small-cell carcinoma, EBRT = external beam radiotherapy, WP = whole-pelvic, CS = central shield, BT = brachytherapy, IC-BT = intracavitary brachytherapy, IC+IS-BT = intracavitary and interstitial brachytherapy, CDDP = cisplatin, NDP = nedaplatin.

Seventy-six patients received chemotherapy, of which 66 received weekly cisplatin administration (40 mg/m^2^ per cycle); 70% (46/66) of the patients received ≥5 cycles (median, 5 cycles; range, 2–6 cycles). Ten patients received monthly nedaplatin administration (80 mg/m^2^ per cycle; range, 1–2 cycles).

### Outcomes

**Figure 1.**
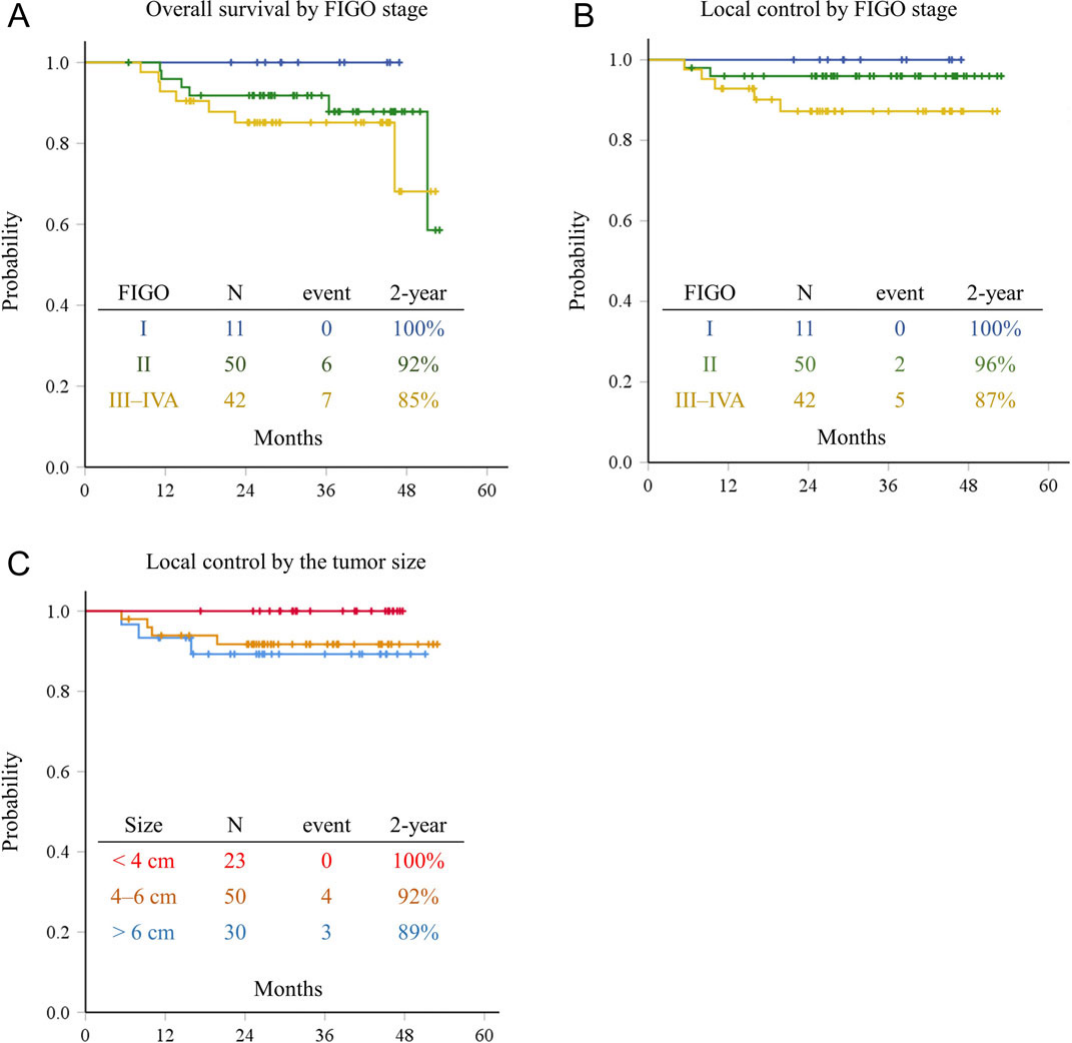
Overall survival rates according to FIGO stage (A), local control rates according to FIGO stage (B), and local control rates according to tumor size (C) in 103 eligible patients.

The median follow-up duration was 31.8 months (range, 6.5–52.9 months). The 2-year OS rate according to stage was 100% in Stage I, 92% in Stage II, and 85% in Stages III–IVA patients, respectively (Fig. 1A). There was no significant difference in the 2-year OS according to stage (*P* = 0.346). The 2-year LC rate according to stage was 100% in Stage I, 96% in Stage II, and 87% in Stages III–IVA patients, respectively, and no significant difference was observed between the groups (*P* = 0.206) (Fig. 1B). After stratification by tumor size at the time of diagnosis, the 2-year LC rates in patients with tumors <4 cm, 4–6 cm and >6 cm were 100%, 92% and 89%, respectively, and no significant difference was observed between the tumor size groups (*P* = 0.307) (Fig. 1C).

**Figure 2.**
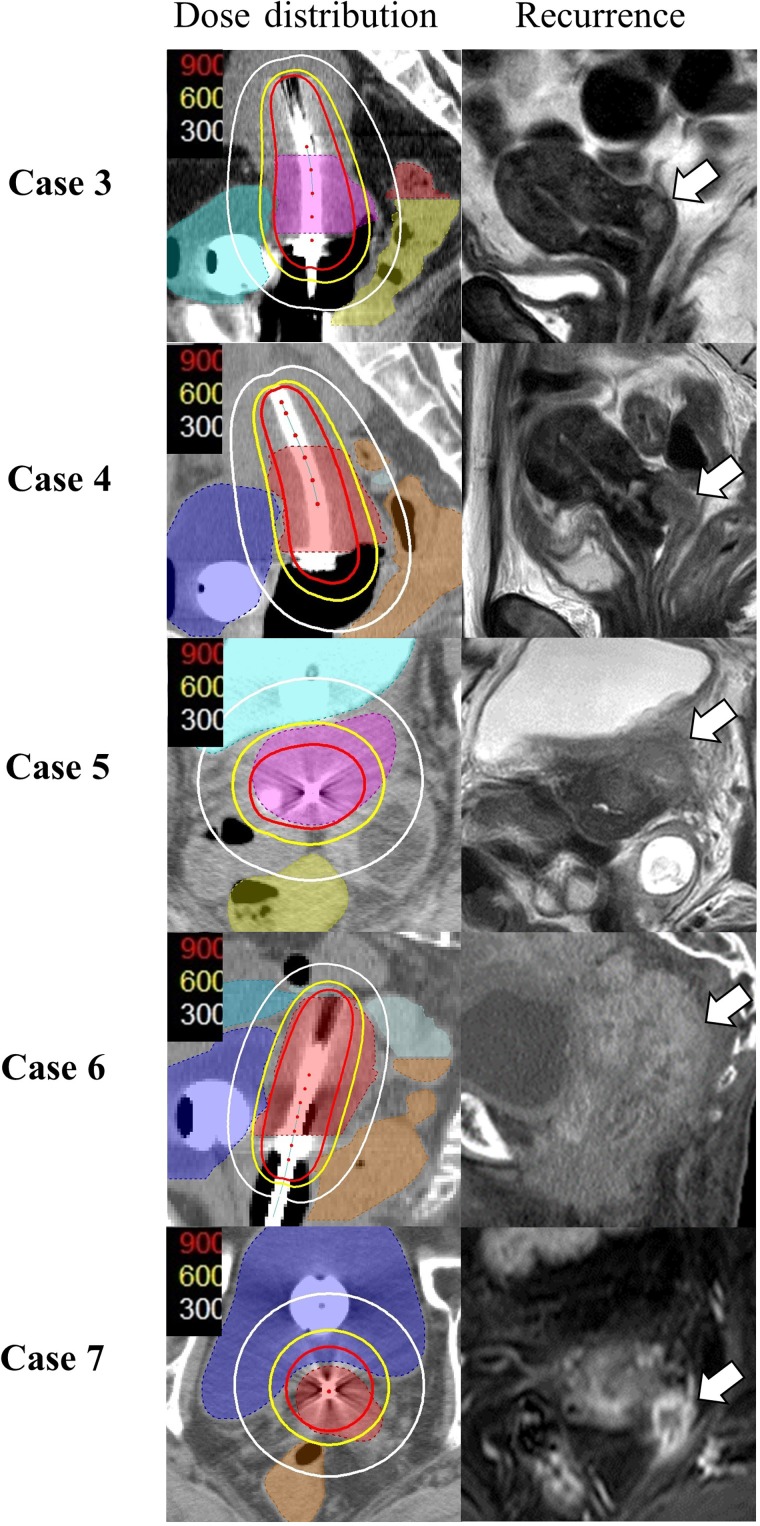
Dose distributions at brachytherapy (BT) and patterns of local recurrence within the primary tumor. HR-CTV of BT sessions only are shown. The white, yellow and red lines represent the 3-, 6- and 9-Gy isodose lines, respectively. White arrows show the sites of local recurrence. Note that the regions of local recurrence were outside the 6-Gy isodose lines in all cases.

Seven patients developed local recurrence (Table 2). Two of the seven patients (Cases 1 and 2) developed recurrence in the lower vagina or vaginal orifice at 5.4 or 15.9 months after treatment. At the beginning of the treatment, no tumor infiltration in these regions was observed in both MRI and gynecological exams. These regions were included in the field of WP-EBRT and were irradiated with 30 Gy and 40 Gy, respectively. However, they were not included in the HR-CTVs for BT and were not irradiated with high-dose BT. Five patients developed recurrence within the extent of the primary tumor at 5.4–19.8 months after treatment. All tumors were included in the HR-CTVs for BT, and cumulative doses of 31.1–41.7 Gy_EQD2_ were delivered to 90% of the HR-CTVs by BT (Fig. 2).

**Table 2.** List of seven patients who developed local recurrence

Patient no.	Age	FIGO stage	Tumor size (mm)	Histology	EBRT WP/CS	BT		Recurrence interval (months)	Recurrence site
						HR-CTV D90 (Gy_EQD2_)	HR-CTV D98 (Gy_EQD2_)		
1	34	IIB	52	SCC	30 Gy/20 Gy	42.4	34.3	5.4	**Lower vagina**
2	77	IIIA	80	SCC	40 Gy/10 Gy	39.7	30.9	15.9	**Vaginal orifice**
3	45	IIB	47	SM	30 Gy/20 Gy	41.7	32.5	9.3	**Cervix**
4	37	IIIB	68	SCC	30 Gy/20 Gy	32.7	27.4	8.0	**Cervix**
5	50	IIIB	103	SCC	40 Gy/10 Gy	31.1	22.6	5.4	**Left parametrium**
6	73	IIIB	45	AD	40 Gy/10 Gy	31.9	23.5	10.0	**Corpus**
7	74	IIIB	44	SCC	40 Gy/10 Gy	35.1	24.4	19.8	**Left parametrium**

SCC = squamous cell carcinoma, SM = small cell carcinoma, AD = adenocarcinoma, EBRT = external beam radiotherapy, WP = whole-pelvic, CS = central shield, BT = brachytherapy.

Late adverse events in the lower gastrointestinal and genitourinary tracts were observed in 30 and 12 patients, respectively, and most were classified as Grade 1 or 2. Two patients developed a Grade 4 rectovaginal fistula, of which one had Stage IVA disease with rectal invasion. Two patients developed a Grade 4 vesicovaginal fistula, of which one had Stage IVA bladder invasion.

### Correlation of clinicopathological factors and DVH parameters with local control

The correlation of LC with various clinicopathological factors and dose–volume parameters was analyzed. The two patients who developed recurrence outside the CTV (Cases 1 and 2) were excluded from the analyses. The factors, including age, T factor, initial tumor size, histology, dose of WP-EBRT, and the use of chemotherapy, were not significantly correlated with LC in univariate analysis using Fisher’s exact test (Table 3).

**Table 3.** Univariate analyses of local recurrence using Fisher’s exact test: recurrence within the HR-CTV (*n* = 5) vs no local recurrence (*n* = 96)

Factors		Local recurrence within the HR-CTV (n)		Total (*n*)	*P*-values
		Presence	Absence		
Age	<50	2	23	25	*P* = 0.595
	≥50	3	73	76	
Tumor size	<60 mm	3	69	72	*P* = 0.623
	≥60 mm	2	27	29	
T factor	T1–2	1	59	60	*P* = 0.155
	T3–4	4	37	41	
Histology	Non SCC	2	12	14	*P* = 0.140
	SCC	3	84	87	
Use of chemotherapy	Presence	5	69	74	*P* = 0.321
	Absence	0	27	27	
WP-EBRT	<35 Gy_EQD2_	2	56	58	*P* = 0.648
	≥35 Gy_EQD2_	3	40	43	
HR-CTV D90WP-EBRT + BT	<72 Gy_EQD2_	4	41	45	*P* = 0.169
	≥72 Gy_EQD2_	1	55	56	
HT-CTV D98WP-EBRT + BT	<63.5 Gy_EQD2_	4	39	43	*P* = 0.160
	≥63.5 Gy_EQD2_	1	57	58	
HR-CTV D90 at BT	<36 Gy_EQD2_	4	10	14	*P* = 0.001
	≥36 Gy_EQD2_	1	86	87	
HR-CTV D98 at BT	<28 Gy_EQD2_	4	12	16	*P* = 0.002
	≥28 Gy_EQD2_	1	84	85	

SCC = squamous cell carcinoma, WP = whole-pelvic, EBRT = external beam radiotherapy, BT = brachytherapy.

**Figure 3.**
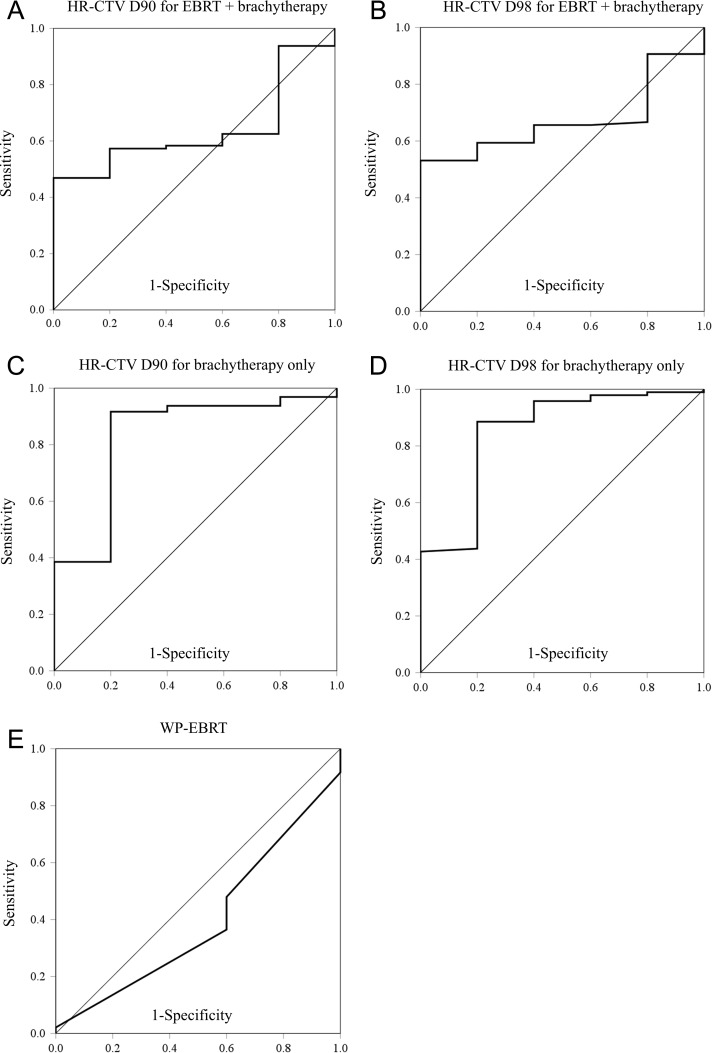
Receiver operator characteristic curve related to local control (LC) based on the sensitivity and specificity of the HR-CTV D90 for external beam radiotherapy (EBRT) and brachytherapy combined (A), HR-CTV D98 for EBRT and brachytherapy combined (B), HR-CTV D90 for brachytherapy sessions only (C), and HR-CTV D98 for brachytherapy sessions only (D).

Regarding dose–volume parameters, we first analyzed the correlation between HR-CTV D90/D98 of the whole WP-EBRT + BT and LC. ROC analyses indicated that the closest points to the ideal coordinates for the HR-CTV D90 and D98 were 72.0 Gy_EQD2_ and 63.5 Gy_EQD2_, respectively (Fig. 3A and B). Fisher’s exact tests using these cut-off values showed that the HR-CTV D90/D98 of WP-EBRT + BT were not significantly correlated with LC (*P* > 0.05) (Table 3). Next, the correlation of the HR-CTV D90/D98 of BT only and LC was analyzed to assess the contribution of BT to LC. ROC analyses showed that the cut-off values for the HR-CTV D90 and D98 at BT were 36.0 Gy_EQD2_ and 28.0 Gy_EQD2_, respectively (Fig. 3C and D). Fisher’s exact tests using these cut-off values showed that the HR-CTV D90/D98 at BT only were significantly correlated with LC (*P* < 0.05) (Table 3).

## DISCUSSION

In the present study, cervical cancer patients were treated with RT or CCRT using CT-based 3D-IGBT. Regarding EBRT, most patients were treated with a combination of 30–40 Gy of WP-EBRT and 20–10 Gy of CS-EBRT. The 2-year OS and LC rates in the present series were favorable, and mostly consistent with those of other series using MRI- or CT-based 3D-IGBT (Table 4). LC rates according to stage or initial tumor size did not differ significantly, suggesting that cervical cancer can be locally controlled irrespective of the stage or tumor size (Fig. 1B and C). Late adverse effects in the rectum and bladder were acceptable. These results suggested that radiotherapy combined with CS-EBRT and 3D-IGBT using the IC or IC + IS technique is an effective treatment modality to control cervical tumors while minimizing late toxicities.

**Table 4.** Reported clinical outcomes of radiotherapy for uterine cervical cancer

Author	Year	Number of patients	Stage (III/IV)	Imaging modality for 3D planning	Local control	Overall survival	Late toxicity ≥Grade 3 for rectum	Late toxicity ≥Grade 3 for bladder
Tan [3]	2005–2007	28	14%/0%	CT	96% (3 years) (Stage I–III)	81% (3 years) (Stage I–III)	1 patient	0%
Pötter [6]	2001–2008	156	24%/4%	MRI	86% (3 years) (Stage IIIB)	45% (3 years) (Stage IIIB)	4% (5 years)	3% (5 years)
Murakami [32]	2008–2010	51	37%/14%	CT	92% (3 years) (Stage I–IV)	82% (3 years) (Stage I–IV)	1 patient	0%
Ohno [31]	2008–2011	80	34%/3%	CT	90% (5 years) (Stage III–IV)	72% (5 years) (Stage III–IV)	0%	1%
Zolciak-Siwinska [8]	2010–2011	216	30%/0.4%	CT	80% (5 years) (Stage III)	52 % (5 years) (Stage III)	4%	3%
Sturdza [7] (retroEMBRACE)	1998–2012	731	23%/3%	MRI	75% (5 years) (Stage IIIB)	42% (5 years) (Stage IIIB)	5% (5 years)	7% (5 years)
Gill [9]	2007–2013	128	16%/0%	CT and MRI	92% (3 years) (Stage I–III)	77% (3 years) (Stage I–III)	1 % (GU/GI toxicity)	
Current study	2013–2015	103	35%/6%	CT	87% (2 years) (Stage III–IV)	85% (2 years) (Stage III–IV)	2% (2 years)	2% (2 years)

GI = gastrointestinal, GU = genitourinary.

To evaluate the treatment of cervical tumors, the GEC-ESTRO working group and ICRU Report 89 provides recommendations regarding several dose–volume parameters, including the HR-CTV D90 and D98 values [14, 15, 29]. There have been several reports on the relationship between dose–volume parameters and local tumor control. Researchers from the Medical University of Vienna demonstrated a positive correlation between HR-CTV D90 and local tumor control [16, 17], and showed that an increased HR-CTV D90 contributed significantly to improving LC in patients with tumors >5 cm in diameter [5, 6]. More recently, a multi-institutional retrospective clinical study demonstrated that HR-CTV D90 was a significant factor for LC of large tumors [7, 18, 30]. These results suggested that dose–volume parameters of HR-CTV were effective predictive factors with respect to local tumor control, and a total HR-CTV D90 dose of >85–87 Gy might be necessary for adequate local tumor control. In these studies, however, patients were treated with a combination of WP-EBRT and BT, and CS-EBRT was not used.

It was recommended that the total dose–volume parameters to the HR-CTV should be estimated by simply adding the biologically equivalent dose of the WP-EBRT and that of every BT session [15]. The CS-EBRT dose is excluded from the estimation. However, phantom studies showed that the contribution of CS-EBRT to the HR-CTV was not negligible, and varied significantly according to tumor size, shape and extent [26, 27]. Furthermore, it is difficult to precisely estimate the contribution of CS-EBRT to the HR-CTV, because the tumor size, shape and position may vary during the treatment course. Therefore, in the present study, we did not estimate dose contribution from CS-EBRT, but estimated the total HR-CTV D90/98 by simply adding those of WP-EBRT and BT using the conventional method. The HR-CTV D90/D98 of WP-EBRT + BT was not significantly correlated with LC in univariate analysis (Table 3). One possible reason for this result is that the cumulative dose of WP-EBRT + BT while omitting the CS-EBRT dose may not represent the dose actually delivered to the HR-CTV when CS-EBRT is used during the treatment course. Another possible reason might be statistical uncertainties due to the small number of local recurrences.

By contrast, the HR-CTV D90 and D98 at BT only were significantly correlated with LC in the present study. Patients who received a cumulative HR-CTV D90 of >36.0 Gy_EQD2_ or a HR-CTV D98 of >28.0 Gy_EQD2_ in the BT sessions had significantly better LC in the univariate analysis (Table 3). The results suggested the importance of BT for local tumor control, and indicated that 90% or 98% of the HR-CTV should be covered with at least 6.5 Gy (~9 Gy_EQD2_) or 5.5 Gy (~7 Gy_EQD2_), respectively, at each BT session. In the five patients who developed local recurrence, the sites of recurrence were outside the 6 Gy isodose line at BT (Fig. 2). Murakami *et al.* and Ohno *et al.* reported similar treatment results using the combination of WP- and CS-EBRT and BT. These authors also indicated that a 6 Gy isodose line should cover the HR-CTV to achieve a HR-CTV D90 of >6 Gy at each BT session [31, 32]. Similarly, sufficient coverage with a high dose to HR-CTV was the most significant prognostic factor in the present study (Table 3, Fig. 2), but the dose of the WP-EBRT was not a significant prognostic factor for local tumor control. Tamaki *et al.* compared the dose distributions of WP- plus CS-EBRT and BT with those of WP-EBRT and BT in their phantom studies, and they also suggested that the low dose volumes in EBRT caused by inserting a CS could be covered with a very high dose of BT, when the BT sources were adequately placed [26, 27]. Therefore, it was suggested from the present study that a HR-CTV D90 of >36.0 Gy_EQD2_ or a HR-CTV D98 of >28 Gy_EQD2_ for all BT sessions might be important factors for local tumor control, when the patients received either 30 or 40 Gy of WP-EBRT.

The optimum method for accurately estimating the dose–volume parameters of the HR-CTV when using CS-EBRT remains to be established. One possible method is to generate composite dose distributions of WP- and CS-EBRT and all BT sessions projected onto one CT series using deformable image registration, and to estimate the cumulative dose–volume parameters for the HR-CTV [33]. However, changes in the uterus and cervix positions and significant regression of the tumor volume may occur during the treatment course, and these positional uncertainties may cause difficulty in generating composite dose distributions. Therefore, further studies are needed to establish the optimum method.

In the present series, two patients developed recurrence in their lower vagina or vaginal orifice. The regions were not included in the respective HR-CTVs at BT because no tumor infiltration was detected in those regions at the beginning of the treatment. Repetitive careful gynecological examinations by both gynecological and radiation oncologists are necessary to check vaginal infiltration [14, 34]. These vaginal recurrences may have reflected vaginal skip metastasis because they were an atypical pattern of recurrence. Positron emission tomography using ^18^F-fluorodeoxyglucose (FDG) in combination with MRI/CT reportedly improve the detection of metastatic lesions, and prognosis in uterine cancer [35, 36], which may help achieve more accurate pretreatment evaluation of cervical tumors.

The present study had several limitations. First, this was a retrospective single-institution study. Second, the small number of cases of local recurrence limited the statistical reliability. However, it is difficult to design a study including a higher number of recurrent cases because of the high LC probability for RT or CCRT in cervical cancer [31, 32].

In conclusion, the present study demonstrated that the dose to the HR-CTV was significantly correlated with local tumor control in RT for cervical cancer, and suggested that optimum dose distribution to cervical tumors by BT particularly contributed to LC. Further multi-institutional clinical studies are necessary to determine the optimum dose–volume parameters of the HR-CTV to sufficiently control the local tumor when using CS-EBRT.
